# The Ability of Extracellular Vesicles to Induce a Pro-Inflammatory Host Response

**DOI:** 10.3390/ijms18061285

**Published:** 2017-06-16

**Authors:** Maike E. van Hezel, Rienk Nieuwland, Robin van Bruggen, Nicole P. Juffermans

**Affiliations:** 1 Department of Intensive Care Medicine, Academic Medical Center, University of Amsterdam, 1012 WP Amsterdam, The Netherlands; m.e.vanhezel@amc.uva.nl; 2 Department of Blood Cell Research, Sanquin Research and Landsteiner Laboratory, 1006 AD Amsterdam, The Netherlands; r.vanbruggen@sanquin.nl; 3 Laboratory of Experimental Clinical Chemistry, Vesicle Observation Centre, Academic Medical Center, 1105 AZ Amsterdam, The Netherlands; r.n.nieuwland@amc.uva.nl

**Keywords:** extracellular vesicles, inflammation, host response

## Abstract

Extracellular vesicles (EVs) can modulate the host immune response, executing both pro- and anti-inflammatory effects. As EVs increasingly gain attention as potential carriers for targeted gene and drug delivery, knowledge on the effects of EVs on the host immune response is important. This review will focus on the ability of EVs to trigger a pro-inflammatory host response by activating target cells. The overall view is that EVs can augment an inflammatory response, thereby potentially contributing to organ injury. This pro-inflammatory potential of EVs may hamper its use for therapeutic drug delivery. Whether removal of EVs as a means to reduce a pro-inflammatory or pro-coagulant response during hyper-inflammatory conditions is beneficial remains to be determined. Prior to any proposed therapeutic application, there is a need for further studies on the role of EVs in physiology and pathophysiology using improved detection and characterization methods to elucidate the roles of EVs in inflammatory conditions.

## 1. Introduction

Extracellular vesicles (EVs), including microparticles and exosomes, are phospholipid-enclosed vesicles of less than 100 nm, diameter often 100-fold smaller than the smallest cells. EVs are released by red blood cells (RBCs), platelets, white blood cells and endothelial cells, and are involved in a broad spectrum of biological activities. Because all cells release EVs into the environment, all body fluids contain high concentrations of cell-derived EVs.

There is increasing evidence that EVs may have specialized functions and play a role in intercellular signaling, for example, by exchanging biomolecules as transmembrane receptors and genetic information [1]. Additionally, EVs may protect cells from the accumulation of intracellular waste. Furthermore, EVs may expose phosphatidyl serine, a negatively-charged phospholipid to which activated coagulation factors can bind, and tissue factor, the protein that initiates coagulation.

During disease states the concentration of EVs, their cellular origin, composition, and functional features may change, thereby affecting disease development and progression. At present, however, the reason why cells release EVs is unknown. As the majority of studies on EVs have been performed in vitro, we can only speculate about the clinical relevance. Furthermore, due to recent improvements in isolation methods of EVs, components previously associated with EVs may need to be reconsidered to be isolation artefacts [2]. Despite these considerable knowledge gaps, endogenously released EVs probably should not be regarded as having only “good” or “bad” effects. To further complicate the interpretation of the relevance of EVs, EVs themselves can also be used by micro-organisms to facilitate spreading and to escape from immune surveillance.

Given the ability of EVs to exchange information between cells, as well as between cells and their microenvironment, the use of EVs in cancer vaccines and drug delivery is increasingly gaining attention. As the natural carrier of signal molecules, EVs may be an attractive vehicle for therapeutic delivery, with a presumed low toxicity. Potential areas of application include cancer [3] and cardiovascular diseases [4]. However, EVs can also modulate the host immune response, executing both pro- and anti-inflammatory effects, which may offset any potential beneficial effects of EVs used as carriers. Thereby, knowledge on the effects of EVs on the host immune response is important. This review will focus on the ability of EVs to trigger a pro-inflammatory host response by activating target cells. In experimental models of hyper-inflammatory disease states, e.g., following ischemia-reperfusion injury, the pro-inflammatory potential of EVs aggravates organ damage. Whether EVs modulate organ damage in patients remains to be determined.

## 2. Inflammatory Effects of Extracellular Vesicles EVs

### 2.1. Extracellular Vesicle (EV)-Produced Inflammatory Mediators

EVs have the ability to modulate the immune system by transferring receptors and inflammatory mediators (Table 1). For example, EVs are essential for the release of interleukin (IL)-1β [5]. Interleukin-1-containing EVs from monocytes activate endothelial cells and stimulate the production of IL-1β from monocytes in an autocrine fashion [6]. In line with this, upon stimulation, macrophages and dendritic cells release vesicles containing IL-1β, caspase-1, and other components of the inflammasome [7]. Furthermore, EVs contain platelet-activating factor [8] and can expose (tumor necrosis factor) TNF receptor 1 [9]. To which extent these inflammatory mediators associated with EVs represent a major fraction of the total amount of such mediators released from cells, however, remains to be shown.

EVs can contribute to an inflammatory response via their lipid fraction, as this fraction can activate the Toll-like receptor (TLR) 4 on macrophages [16]. The ability of EVs to activate TLR4 is impaired after incubation with an inhibitor of phospholipase D, confirming that the lipid component of EVs may contribute to the inflammatory response. In addition, EVs from dendritic cells, macrophages and plasma contain enzymes involved in synthesis of leukotrienes [17]. Additionally, RBC-derived EVs can promote the secretion of the von Willebrand factor by endothelial cells [13], which is a plasma ligand for cell adhesion. An increase in the von Willebrand factor may thereby promote cellular adhesion and augment inflammation. In vitro, it was shown that RBC-derived EVs can interact with platelets to increase inflammatory chemokine bioavailability [30]. Taken together, EVs can exert direct pro-inflammatory effects by the production of pro-inflammatory mediators, without the interaction of immune cells.

### 2.2. EV-Mediated Pro-Inflammatory Responses of Effector Cells in the Circulation

EVs can directly interact with different cell types, inducing a functional inflammatory response from immune cells. Incubation of whole blood with RBC-derived EVs induced a dose-dependent production of (TNF)α, IL-6, and IL-8 [10]. The response of cells to EVs was very strong, equaling the response to incubation with TNFα. EVs can directly interact with various types of cells.

RBC-derived EVs can dose-dependently trigger human neutrophils to increase production of CD11b [31], induce a respiratory burst, and increase the ability of neutrophils to phagocytose. Similar results were obtained in a murine model, in which injection of RBC-derived EVs into healthy mice primed neutrophils, as reflected by an increased expression of neutrophil CD11b [11]. RBC-derived EVs can also have a direct interaction with monocytes. Using confocal microscopy, it was shown that RBC-derived EVs are phagocytized by monocytes, a process that is partially inhibited by incubation with antibodies directed against complement receptor 3.

Monocyte-derived EVs also have largely pro-inflammatory effects, mostly through interaction with endothelial cells, but also with other cells, including monocytes themselves, fibroblasts, and smooth muscle cells [14]. Monocyte-derived EVs interact with the endothelium, inducing expression of adhesion molecules [6] as well as nitrosative stress [15]. Monocyte-derived EVs can induce production of pro-inflammatory mediators MCP-1 and IL-6 from podocytes, associated with increased levels of a marker of glomerular permeability, indicating glomerular inflammation [32]. Additionally, monocyte-derived EVs containing caspases are capable of inducing apoptosis in endothelial cells [33] and in vascular smooth muscle cells [34].

Neutrophil-derived EVs are the least studied EVs to date. It is clear that levels of circulating neutrophil-derived EVs are increased under inflammatory conditions, which holds true for auto-immune conditions, asthma, and severe infections [35,36]. Neutrophil-derived EVs can mediate binding of neutrophils to red blood cells following complement activation [20]. T and B cell-derived EVs have been mostly described for their interaction with tumor cells, although pro-inflammatory effects of T cells have been demonstrated [21,22].

Platelet-derived EVs have mostly been implicated in coagulation, but can also exert pro-inflammatory effects. Platelet-derived EVs from synovial fluid from patients with rheumatoid arthritis increased production of inflammatory cytokines in fibroblast-like synoviocytes in an IL-1-dependent manner [37]. Staphylococcal-induced formations of platelet-EVs were also able to stimulate monocytes to produce IL-1β, TNFα, and MCP-1 [38]. However, platelet-derived EVs also have anti-inflammatory effects. T cells exposed to platelet-derived EVs had reduced the production of interferon γ (IFNγ), TNFα, and IL-6 secretion [39]. Other pro-inflammatory effects of platelet-derived EVs were recently summarized [40].

### 2.3. EV-Mediated Inflammatory Responses of Endothelial Cells

EVs activate endothelial cells via different pathways. Neutrophil-derived EVs trigger endothelial cells to secrete IL-6 and to produce tissue factor (TF) [19]. Monocyte-derived EVs activate endothelial cells, reflected by an increase in the release of EVs and an increase in production of adhesion markers, thus amplifying the inflammatory process [14]. In contrast, RBC-derived EVs alone do not induce the production of endothelial adhesion markers, but when endothelial cells are incubated with RBC-derived EVs in the presence of monocytes, expression of ICAM-1 and E-selectin increase compared to endothelial cells incubated with EV-depleted RBCs [13]. RBC-derived EVs do not activate endothelial cells when co-incubated with neutrophils, showing that the effect is specific for monocytes.

Additionally, platelet-derived EVs activate endothelial cells. These EVs contain arachidonic acid, which is transferred to the endothelial cells and then promotes the production of cyclooxygenase-2 and adhesion molecules [23,41]. In an in vitro model of lipopolysaccharide (LPS)-stimulated endothelial cells, the addition of platelet-derived EVs increased endothelial activation and induced the respiratory burst in neutrophils [24]. Furthermore, platelet-derived EVs also modulate interactions between monocytes and endothelial cells. The interaction between EVs and the endothelium may be amplified by the release of pro-inflammatory cytokines [42] and platelet activating factor [23] from endothelial cells. In turn, endothelial-derived EVs further activate the endothelium, thus further perpetuating the inflammatory response [29]. Taken together, EVs seem to mediate increased adherence of immune cells to the endothelium with the induction of a pro-inflammatory response.

### 2.4. EV-Mediated Inflammatory Responses in Effector Cells in Tissues

EVs in body fluids or tissues can modulate the local inflammatory responses. During rheumatoid arthritis, EVs from synovial fluid trigger the production of pro-inflammatory mediators by synovial fibroblasts, via the transfer of arachidonic acid from leukocytes to fibroblasts, thereby contributing to the destructive activity of fibroblasts [43]. In adipose tissue, EVs from adipocytes increased CD16 and CCR5 production on monocytes, thereby inducing monocyte migration into the adipose tissue, contributing to the chronic inflammatory phenotype in obesity [44]. In a model of traumatic brain injury, microglial-derived EVs are released into the circulation [45]. These EVs initiated neuroinflammation after injection into the cortex of healthy animals. In addition, EVs derived from monocytes also promote inflammation by interaction with endothelial cells, monocytes, fibroblasts, and smooth muscle cells [14]. Taken together, there is increasing evidence suggesting that EVs from different cellular sources act in concert with the endothelium in inducing and perpetuating inflammatory host responses.

### 2.5. EV-Mediated Pro-Coagulant Response

One of the first identified roles of EVs has been its participation in coagulation. RBC-derived EVs can expose PS, a negatively-charged phospholipid to which (activated) coagulation factors can bind, thereby promoting thrombin generation [1]. Another pathway by which RBC-EVs may promote coagulation is that these EVs may be a source of von Willebrand factor [13]. Endothelial- and monocyte-derived EVs may also support coagulation by the expression of tissue factor (TF). The amount of TF bearing monocyte- and platelet-derived EVs have been related to infarction severity in patients [46]. Additinoally, in cancer patients, the levels of tumor-derived EVs exposing TF are associated with the development of venous thromboembolism, although cause and effect are unclear [47]. Together, in patients with an increased risk, EVs are associated with thrombotic events.

Increased coagulation activation is a hallmark of a pro-inflammatory response. The endothelium undergoes pro-thrombotic changes and platelets become activated, exhibiting adhesive properties. In diffuse intravascular coagulation (DIC), there is formation of microthrombi with the consumption of platelets and coagulation factors, associated with adverse outcome [48]. EVs probably also play a role in the hyper-coagulative response in inflammation. In human endotoxemia, the levels of total EVs and platelet-derived EVs are increased, bearing TF, showing increased thrombin generating activity [25]. In patients with septic shock, the systemic levels of TF-exposing EVs are increased [49]. Additionally, in severe sepsis patients, endothelial-derived EVs are associated with the occurrence of DIC [50], suggesting that EVs play a role in clinically-relevant coagulation disorders in sepsis. EVs bearing TF derived from tissues also contribute to local inflammation. In patients with acute lung injury, the concentrations of TF-bearing EVs in the lung are higher compared to controls [51]. Furthermore, in patients with sterile inflammation due to arthritis, TF-exposing EVs are present in joint fluid of inflamed joints [52]. Taken together, EVs can promote coagulation and are often pro-coagulant. In particular, platelet-derived EVs are associated with thrombotic events in patients at risk, whereas EVs from all sources can contribute to inflammation-induced coagulation.

From a therapeutic point of view, the role of EVs in disease states characterized by a reduced ability to clot is also interesting. Under these conditions, the amount and activity of EVs seem to correlate with the ability to clot. In coagulopathic bleeding trauma patients, the levels of platelet-derived EVs were lower compared to minimally-injured control patients, and were associated with reduced thrombin generating capacity [53]. Given that the levels of RBC- and leukocyte-derived EVs did not differ between groups, it has been suggested that in particular decreased levels of platelet-derived EVs may contribute to an anti-coagulant phenotype. Findings in a murine model of traumatic brain injury (TBI) also underline that low levels of circulating platelet-derived EVs are associated with anti-coagulant activity [54]. Mice showed impaired clot formation following head injury, which was partially restored by the addition of platelet-derived EVs from healthy donor mice to the blood of the mice who had sustained TBI. Whether platelet-derived EVs could be a potential therapeutic target to modulate coagulation responses remains to be determined.

### 2.6. EV-Mediated Host Response in Inflammatory Disease States

In most models of sterile inflammation, EVs seem to perpetuate organ injury. In a mouse model of hepatic ischemia-reperfusion injury, endothelial cells, platelets, and T cells release EVs into the circulation, containing F2-isoprostanes, indicating oxidative damage to membrane lipids [18]. In particular, EVs from macrophages and platelets were able to trigger TNFα release from hepatocytes, thereby perpetuating hepatic injury, a process that is ROS-dependent. Similarly, in a mouse model of hemorrhage and resuscitation, injection of RBC-derived EVs increased pulmonary neutrophil accumulation and histologic evidence of lung injury compared with mice resuscitated without EVs [11]. Additionally, in an endotoxemia mouse model, injection of RBC-derived EVs aggravated leukocyte recruitment to the lungs and increased levels of proinflammatory cytokines, which was abrogated in C5aR-deficient mice, suggesting that RBC-derived EVs are pro-inflammatory via complement activation [12].

In sepsis, the contribution of EVs to inflammation is probably more complicated. EVs from different cellular origin may play a role at multiple sites, as both the activated endothelium releases EVs which may communicate with immune cells, and EVs shed from immune cells interact with the endothelium. As discussed above, the levels of circulating EVs from platelets, leukocytes, and endothelial cells are increased in sepsis. EVs promote the adhesion of platelets and/or leukocytes to the endothelium, thus playing the role of trigger in the production of pro-inflammatory cytokines such as IL-1β, IL-8, and TNFα [41]. Of note, it is not only EVs from the host immune response that drive the inflammatory response in sepsis. In vitro, staphylococcal super antigen-like protein 5 (SSL5) from *Staphylococcus aureus* dose-dependently induced the generation of platelet-derived EVs, which then bind to monocytes, causing platelet aggregation and release of inflammatory mediators IL-1β, TNFα, and MCP-1 [38]. These EVs induced by *S*. *aureus* also enhanced monocyte migration. Taken together, there seems to be a role for EVs in perpetuating the inflammatory response in sepsis.

EVs play an important role in trauma, a state which is also characterized by a hyper-inflammatory host response with activated endothelium, together with immune suppression, as demonstrated by a markedly reduced ability of immune cells to generate a pro-inflammatory response. Circulating EVs may have several functions. Low levels of platelet-derived EVs correlate with disturbed coagulation potential in trauma, as described before. In addition, EVs regulate the host response in trauma. The reduced immune response towards endotoxin challenge in trauma is associated with low levels of circulating EVs in trauma compared to controls [26]. The host response to endotoxin was restored by the addition of EVs, suggesting that these EVs drive the synthesis of pro-inflammatory cytokines in response to LPS [26]. Most circulating endogenous EVs are derived from platelets, suggesting that platelets are the most important source of EVs involved in host response post-injury. Whether exogenously-added EVs could boost an immune response in trauma remains to be determined.

### 2.7. EVs also Have Anti-Inflammatory Effects

The anti-inflammatory effects are not the focus of this review and are, therefore, not discussed in detail. However, in the discussion that pro-inflammatory effects from EVs may be hurdle from a therapeutic viewpoint, it needs to be acknowledged that EVs can also exert anti-inflammatory effects, which seem to be primarily mediated by neutrophil-derived EVs. Neutrophil-derived EVs induce downregulation of the transcription of pro-inflammatory cytokines [55] and the release of stored TGF-β1 [56] from macrophages. Leukocyte-derived EVs isolated from sepsis patients increased the phagocytic capacity of cells [14]. Similarly, neutrophil-derived EVs from sepsis patients induced monocyte phagocytosis ex vivo [57]. In an infectious sepsis mouse model, injection of leukocyte-derived EVs had anti-inflammatory effects associated with immune dysfunction, characterized by an increased bacterial load, decreased neutrophil recruitment, increased expression of IL-10 and a negative effect on mortality [35]. Furthermore, platelet-derived EVs have anti-inflammatory effects. T cells exposed to platelet-derived EVs had reduced production of interferon γ (IFNγ), TNFα, and IL-6 secretion [39]. Other anti-inflammatory effects of platelet-derived EVs were recently summarized [40].

## 3. Conclusions

EVs act by triggering signaling pathways and exchanging information, and thereby have gained interest as carriers for targeted drug delivery. When contemplating the use of EVs as carriers, it should be taken into account that EVs can augment an inflammatory response, thereby contributing to organ injury. Even though it should be noted that most of these effects of EVs have been studied in vitro and the clinical relevance of these findings is highly unclear, this pro-inflammatory potential of EVs may hamper its use as carriers in various conditions, which may particularly hold true for sterile inflammatory conditions.

Removal of EVs as a method to reduce a pro-inflammatory response during hyper-inflammatory conditions, may be an alternative potential beneficial intervention. When considering the pro-coagulant response, the association between platelet-derived EVs and DIC in sepsis seems a consistent finding throughout the studies. Whether these EVs could be removed, e.g., by filtering, to treat DIC in sepsis without increasing the risk of bleeding is, however, undecided.

The use of EVs to restore an inhibited host response is another intriguing potential application. In this concept, the timing of intervention is probably crucial. Sepsis is characterized by both hyper-inflammation, as well as by immune tolerance, i.e., the inability of immune cells to respond to endotoxins. These conditions occur simultaneously but probably not in a balanced fashion, and also with a highly variable phenotype between patients [58]. Additionally, trauma is characterized by a pro-inflammatory response coinciding with a decreased ability to respond to bacterial antigens. In trauma patients, addition of EVs has been shown to restore cellular reactivity to LPS in an ex vivo design [26]. Whether EVs can be used to “boost” the immune system during a state of reduced host immune response needs further investigation.

Taken together, EVs have clearly entered the scene. Since these vesicles are small and difficult to study [2], recent and still ongoing efforts to improve isolation, detection, and characterization will further elucidate their roles in physiology and pathology.

## Figures and Tables

**Table 1:** Pro-inflammatory effects of endogenous extra-cellular vesicles from immune cells and endothelial cells.

Cellular EV Origin	Target Cell	Inflammatory Effect	Type of Study	Reference
RBC	Whole blood	Production of TNFα, IL-6, IL-8	Ex vivo	Straat [10]
RBC	Granulocytes	Respiratory burst	In vitro, in vivo	Belizaire [11]
RBC	–	Leukocyte homing	In vivo	Zecher [12]
RBC	Monocytes	Binding and phagocytosis	In vitro	Straat [13]
RBC	Endothelial cells	Expression of ICAM-1, E-selectin	In vitro	Straat [13]
Monocyte	Monocyte	IL-1β production	In vitro	McKenzie [5]
Monocyte	Endothelial cells	IL-1β production	In vitro	Wang [6]
Monocyte	Endothelial cells	Expression of ICAM-1, VCAM-1, E-selectin	In vitro	Wang [6], Halim [14]
Monocytes	Endothelial cell	Induction nitrosative stress	In vitro	Mastronardi [15]
Macrophages	Macrophages	Activate TLR-4, TNF production	In vitro	Thomas [16]
Macrophages	–	IL-1, caspase-1 production	In vitro	Qu [7]
Marcophages, Dendritic cells	–	Leukotrienes synthesis, Granulocyte migration	In vitro	Esser [17]
Macrophages	Hepatocytes	TNF production	In vitro	Teoh [18]
Granulocytes	–	PAF production	In vitro	Watanabe [8] Mesri [19]
Granulocytes	Endothelial cells	TF and IL-6 production	In vitro	Mesri [19]
Granulocytes	Red blood cells	Complement activation	In vitro	Gasser [20]
T cells	Monocytes	TNF, IL-6 production	In vitro	Scanu [21]
T cells	Endothelial cells	No synthase, COX-2 production	In vitro, in vivo	Martin [22]
Platelets	Endothelial cells	COX-2 production	In vitro	Barry
Platelets	Endothelial cells	PAF production	In vitro	Wolf [23]
Platelets	Endothelial cells	CD11b expression	In vitro	Xie [24]
Platelets	–	Thrombin generation	In vivo	Mooberry [25]
Platelets	Whole blood	Production IL-6, TNFα	Ex vivo	Balvers [26]
Endothelial cells	Endothelial cells	Transfer miRNA	In vitro	Jansen [27]
Endothelial cells	Endothelial cells	Adherence monocytes, expression of ICAM-1	In vitro	Lee [28]
Endothelial cells	Endothelial cells	IP-10 production	In vitro	Liu [29]

EV: extracellular vesicle, TNF: tumor necrosis factor, TF: tissue factor, IL: inter-leukin, ICAM: intercellular adhesion molecule, VCAM: vascular cell adhesion molecule, TLR: Toll-like receptor, PAF: platelet activating factor.
